# CMV Infection Risk Factors and Viral Dynamics After Valganciclovir Prophylaxis: 10 Years of Experience in Lung Transplant Recipients

**DOI:** 10.3390/microorganisms12112360

**Published:** 2024-11-19

**Authors:** Sarela García-Masedo Fernández, Rosalía Laporta, Christian García Fadul, Myriam Aguilar Pérez, Jorge Anel Pedroche, Raquel Sanabrias Fernández de Sevilla, Ana Royuela, Isabel Sánchez Romero, María Piedad Ussetti Gil

**Affiliations:** 1 Microbiology Department, Hospital Universitario Puerta de Hierro, 28222 Majadahonda, Spain; 2 Pneumology Department, Hospital Universitario Puerta de Hierro, 28222 Majadahonda, Spainpied2152@separ.es (M.P.U.G.); 3 Pharmacy Department, Hospital Universitario Puerta de Hierro, 28222 Majadahonda, Spain; 4 Clinical Biostatistics Unit, Instituto de Investigación Sanitaria Puerta de Hierro-Segovia de Arana, 28222 Madrid, Spain

**Keywords:** cytomegalovirus, lung, transplant, valganciclovir, prophylaxis, discontinuation

## Abstract

(1) The prevention of cytomegalovirus (CMV) in lung transplant recipients (LTx) is based on the administration of VGC for a period of 6–12 months, but there is little information on the premature discontinuation of the drug. Our objective was to evaluate the reasons for early cessation of VGC and the dynamics of CMV replication after discontinuation. (2) We carried out a retrospective study of LTx on VGC prophylaxis according to guidelines, with an outpatient follow-up period of >90 days. The detection of any level of CMV-DNA in the plasma (Cobas, Roche Diagnostics^®^) during a period of 18 months after the discontinuation of VGC was considered positive. (3) We included 312 patients (64% male, mean age 53.50 ± 12.27; 71% D+R+, 15% D−R+, and 14% D+R−) in our study. The prescribed prophylaxis was completed by 179 patients (57.05%). The mean duration of prophylaxis was 7.17 ± 1.08 months. The recorded reasons for VGC discontinuation in 133 patients (43%) were myelotoxicity (n = 55), impaired renal function (n = 32), and gastrointestinal disturbances (n = 11). The reason for discontinuation was not recorded for 29 patients. CMV-DNA was detected in 79% (n = 246) of cases, and D+R+ and D+R− recipients showed a high risk of detection (*p* < 0.001). The median times to onset of CMV-DNA detection were 35 days in D+R−, 73 days in D+R+, and 96 days in D−R+ (*p* < 0.001). (4) Adverse effects of VGC are frequent in LTx. CMV-DNA detection is very common after the discontinuation of VGC and is related to the CMV donor and recipient serostatus.

## 1. Introduction

Cytomegalovirus (CMV) infection is a common cause of morbidity and mortality in lung transplant recipients (LTx). Following initial infection, CMV may remain latent in CD34+ myeloid progenitors and CD14+ monocytes or produce invasive disease in immunocompromised individuals, such as LTx [1,2,3,4,5].

The risk of infection depends mainly on the donor–recipient serostatus at the time of transplantation; therefore, recipients are classified as high-risk (D+R−), intermediate-risk (R+), or low-risk (D−R−).

CMV infection usually occurs within the first year after transplantation. The severity of infection ranges from asymptomatic to a viral syndrome or invasive disease in the lung parenchyma, gastrointestinal tract, or retina. Without preventive therapy, CMV disease (CMVD) affects more than 50% of D+R− recipients [6], with rates up to three times higher than those observed in R+ ones [7]. Apart from invasive disease, CMV could cause a number of indirect effects on graft and patient survival as a result of the interaction of the virus with the host immune system. These effects are independent of the detected levels of viremia and may be related to low levels of viral load over prolonged periods of time. The indirect effects associated with CMV include acute and chronic graft rejection, atherosclerosis, post-transplant diabetes mellitus (DM), and an increase in opportunistic infections [8,9].

In order to prevent CMV infection, international guidelines recommend the prophylactic administration of ganciclovir (GCV) followed by valganciclovir (VGC) during the highest-risk period in the first months post-transplant. Guidelines recommend universal prophylaxis with VGC for D+R− LTx for twelve months and consider continuation with pre-emptive therapy depending on the individual patient’s risk. For R+ LTx, guidelines recommend 6 months of VGC, followed by pre-emptive therapy for another 6 months [10].

Viral replication is very frequent after prophylaxis. This may be due to the virostatic effect of VGC and its lack of action on latent viruses in infected cells [11]. The risk of infection is particularly high in LTx as a result of the high levels of immunosuppression and the high number of lymphoid cells and macrophages with latent or replicating viruses in the graft [6,12,13].

The implementation of long-term prophylaxis strategies has led to a significant reduction in the incidence of infection and invasive disease [14]. However, according to data from the International Registry of the International Society for Heart and Lung Transplantation (ISHLT), despite the widespread use of antivirals, such as GCV/VGC, high-risk serostatus (D+R−) remains a risk factor for medium- and long-term mortality [15].

The long-term administration of VGC may be associated with a higher likelihood of premature discontinuation due to adverse effects, particularly myelotoxicity. Discontinuation rates of up to 50% have been reported in solid organ transplant recipients [16]. In LTx, the risk of discontinuation can be up to 20 times higher compared to in other solid organ transplants [17]. Letermovir, a new antiviral drug with fewer side effects, has been approved by both the EMA and the FDA for CMV prophylaxis in high-risk kidney transplant recipients. However, there are no prospective clinical trials supporting its use in lung transplant recipients. Therefore, despite the potential adverse effects and the risk of early discontinuation, VGC remains the first-line agent recommended by international guidelines for CMV prophylaxis in LTx [10].

The discontinuation of prophylaxis in LTx is particularly concerning, and the cessation of VGC due to side effects like neutropenia can lead to an increased risk of CMV reactivation and associated complications. Few studies have examined the degree of adherence to the VGC prophylaxis guidelines in LTx. The reasons for its discontinuation, the consequences of early prophylaxis termination, and its impact on viral dynamics in lung transplant recipients are also yet to be thoroughly described [14,18].

The aim of this study was to analyze the degree of compliance with VGC prophylaxis in lung transplant recipients, the reasons for early cessation, the frequency of detection of CMV-DNA in the plasma after the discontinuation of prophylaxis, and the risk factors associated with its development.

## 2. Materials and Methods

### 2.1. Study Design and Patient Population

We designed an observational, retrospective, and analytical study that included all patients aged ≥18 years who underwent lung transplantation at Hospital Universitario Puerta de Hierro Majadahonda (Madrid, Spain) between 1 January 2009 and 31 December 2019, and who received sequential antiviral prophylaxis against CMV with GCV/VGC with a postoperative outpatient follow-up period of ≥90 days in our pulmonology department. The study protocol was approved by the Ethics Committee of our center on 20 November 2023 (no. 196/23). Prior to the collection of data, patients were required to provide written informed consent.

The follow-up period was 18 months after the discontinuation of prophylaxis. Patients without prophylaxis (low-risk recipients (D−R−)) and patients on therapeutic doses of GCV/VGC for immediate post-transplant viral replication or lung retransplantation were excluded.

Our standard approach to immunosuppression includes baliliximab and a life-time triple immunosuppression regimen with tacrolimus, mycopnenolate mofetil, and prednisone.

The protocol of antimicrobial prophylaxis includes nebulized Ambisome (6 mL weekly) and trimethoprim–sulamethoxazole three times a week.

The electronic medical records and laboratory values recorded were demographic data, underlying disease, type of transplant, donor and recipient CMV serology, start and end date of prophylaxis, reason for interruption, date of the first viral load, and maximum viral load (IU/mL).

### 2.2. Antiviral Prophylaxis and Monitoring of CMV Infection or Disease

High-risk patients (D+R−) received prophylaxis with intravenous (IV) GCV (5 mg/kg/24 h) for the first week post-transplant, followed by oral VGC (900 mg/24 h) for the first 12 months. Prophylaxis in these patients was complemented by IgG-CMV (Cytotec^®^) at 100 mL per dose every 48 h for the first week, weekly for the first month, and then monthly for the first year.

Patients at an intermediate risk of CMV (D−R+ or D+R+) received IV GCV (5 mg/kg/24 h) during the first week post-transplant, followed by oral VGC (900 mg/24 h) for the first six months.

The GCV and VGC dosages were adjusted according to the glomerular filtration rate in all cases. In patients with leukopenia and/or plateletopenia, drug administration was delayed or discontinued. According to the patient’s serostatus, IgG-CMV (Cytotec^®^) was initiated or maintained, with PCR monitoring of the plasma CMV-DNA twice weekly in hospitalized patients or once weekly in outpatients. Immunosuppression management during neutropenia was based on the patient’s characteristics and the balance between the risk of rejection and infection. Mycophenolate was reduced or temporarily discontinued, while the usual doses of tacrolimus and corticosteroids were maintained. The criteria for prescribing granulocyte colony-stimulating factors (G-CSFs) were based on the severity of neutropenia, the patient’s characteristics, and the physician’s criteria.

After stopping prophylaxis, plasma CMV-DNA monitoring was performed weekly for a period of 8–12 weeks depending on the patient’s risk serostatus and the physician’s discretion.

The plasma CMV viral load was measured from January 2009 to April 2017 using the COBAS Ampliprep/COBAS Taqman CMV Test (Roche Diagnostics^®^, Basel, Switzerland), with the results reported as copies/mL (lower limit of quantification (LLoQ) of 150 copies/mL). Starting in April 2017, viral load was measured with COBAS 6800 and reported in international units (IU)/mL (LLoQ: 34.5 IU/mL) according to the WHO International Standard for Human CMV for Nucleic Acid Amplification Technique (National Institute for Biological Standards and Controls, NIBSC 09/162). The conversion factor between copies/mL and IU/mL of CMV-DNA was 1.1 copies/mL (0.91 IU/copy), calculated according to the WHO International Standard (NIBSC 09/162). Any level of CMV-DNA detected in the plasma was considered positive.

### 2.3. Definitions

Patients who discontinued prophylaxis were defined as patients who did not complete the full duration of antiviral prophylaxis as recommended by international guidelines.

CMV-DNA detection was defined as the detection of DNA by PCR assay in samples of plasma. Any level of CMV-DNA plasma detection in an 18-month follow-up period after the discontinuation of VGC was considered positive.

Time to CMV-DNA detection was defined as the time to the first instance of CMV-DNA detection after the discontinuation of VGC.

High-level CMV viral load was defined as a CMV viral load more than or equal to 1000 IU/mL. Low-level CMV viral load was defined as being between 35 IU/mL and 999 IU/mL.

Neutropenia was defined as a peripheral blood neutrophil count of <1500/L.

Impaired renal function was defined as a significant decrease in kidney function, evidenced by an increase in serum creatinine levels (>0.9 mg/dL) or a decline in glomerular filtration rate (GFR < 60 mL/min).

Gastrointestinal side effects were defined as a range of symptoms including nausea, vomiting, diarrhea, or abdominal pain.

Chronic graft allograft dysfunction (CLAD) was defined as a substantial and persistent decline (≥20%) in the measured FEV 1 value from the reference (baseline) value. The baseline value is computed as the mean of the two best postoperative FEV 1 measurements (taken >3 weeks apart) [19].

### 2.4. Statistical Analysis

Descriptive analysis was performed on all recorded variables. Pearson’s chi-square test was used for the analysis of variables associated with the first instance of CMV-DNA detection. For quantitative variables, both Student’s *t*-test and non-parametric tests (Mann–Whitney U-test or Kruskal–Wallis) were used if the distributions of the variables did not meet the assumptions of normality. The normality assumption was tested through the Shapiro–Wilk test. Logistic regression models were used to identify the factors associated with CMV-DNA detection, and the effect size was expressed as the odds ratio (OR) and corresponding 95% confidence interval.

A competitive risk analysis was performed to study the time to the first instance of CMV-DNA detection, in which the primary event was the first instance of CMV-DNA detection, and patient death without it was considered a competing event. The cumulative incidence function (CIF) was used to estimate the time to CMV-DNA detection for the different risk groups. A competitive risk regression analysis using the Fine–Gray method was performed to identify the impact of donor–recipient serology on the probability of CMV-DNA detection. Regression coefficients were expressed as the subhazard ratio (SHR) with their respective 95% confidence intervals (CIs).

For all analyses, the significance level was defined as a *p* value below 0.05. Statistical analysis was performed using Stata version 18.0 (StataCorp. 2023. Stata Statistical Software: Release 18. College Station, TX, USA: StataCorp LLC).

## 3. Results

### 3.1. Characteristics of the Study Population

**Figure 1:**
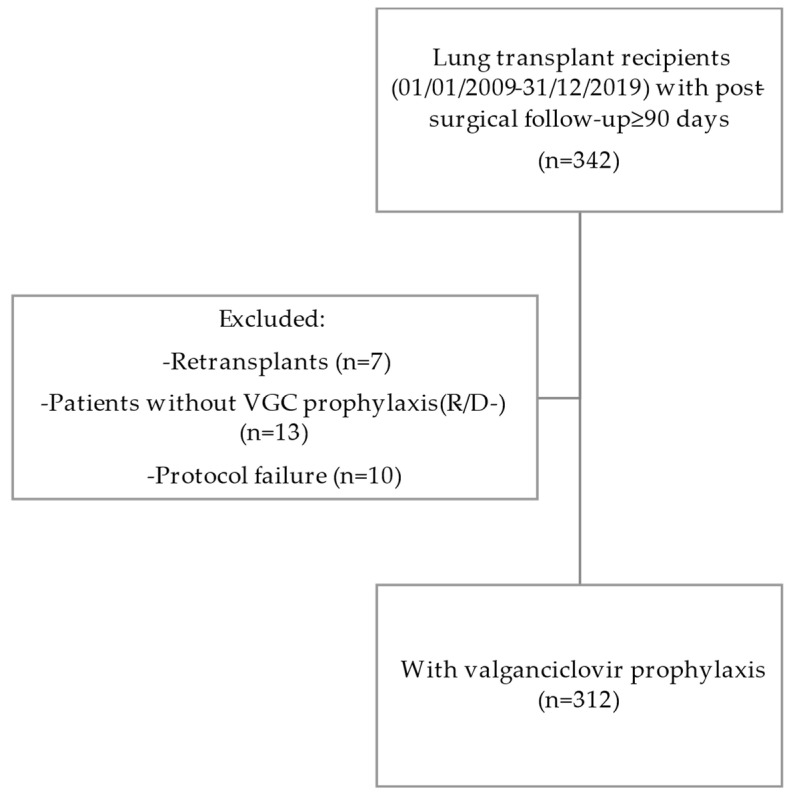
Flow chart of patients under study.

A total of 312 lung transplant recipients who received VGC prophylaxis between 2009 and 2019 and underwent post-transplant follow-up for at least 90 days in our pulmonology department were included (Figure 1).

The baseline characteristics are listed in Table 1. The mean age of the patients at the time of transplantation was 53.50 years (range: 16–68). Most patients were D+R+ (71%), followed by D−R+ (15%) and D+R− (14%). Significant age differences were observed between the groups (*p* < 0.001). Sixty-four percent of the patients were men, and the most frequent underlying diseases were chronic obstructive pulmonary disease (COPD), diffuse interstitial lung disease (ILD), and cystic fibrosis. The majority of the recipients underwent a double lung transplant (79%).

### 3.2. Compliance with CMV VGC Prophylaxis

The prescribed prophylaxis was completed by 179 patients (57.05%). The demographic characteristics of the patients in terms of VGC discontinuation are described in Table 2. The mean duration of GCV/VGC administration was 7.18 ± 4.08 months (mean ± SD). The D+R− patients received VGC for a longer period of time than the R+ ones (D+R−: 8.8 ± 5.5 months, D+R+: 7.01 ± 3.85 months, and D−R+: 6.55 ± 3.3 months; *p* < 0.01).

Out of the 133 patients who did not complete the total recommended duration of prophylaxis, 127 discontinued due to the development of adverse effects (95%): 55 patients due to neutropenia, 32 due to impaired renal function, and 11 due to gastrointestinal side effects. The reason for discontinuation was not recorded in the medical reports of 29 cases. Prophylaxis was also discontinued in six asymptomatic patients due to viral shedding episodes, which were treated with full doses of VGC.

The D+R− recipients had significantly lower compliance rates compared to the R+ ones (D+R−: 30%, D−R+: 58%, and D+R+: 62%; *p* = 0.003). No significant differences in discontinuation rates were observed based on age (*p* = 0.088), sex (*p* = 0.379), underlying disease (*p* = 0.054), or type of transplant (*p* = 0.807) (Table 2).

### 3.3. CMV-DNA Detection

During the follow-up period, CMV-DNA plasma was detected in 246 out of the 312 patients included (79%). The demographic characteristics in relation to CMV-DNA detection are described in Table 3.

The peak CMV-DNA levels were ≤10³ IU/mL in most cases (n = 176: 72%). Of these, 136 patients (77%) had levels within the range of ≥35 IU/mL to 250 IU/mL, 24 (14%) had levels between 251 IU/mL and 500 IU/mL, and 16 (9%) had levels between 501 IU/mL and 10³ IU/mL.

CMV-DNA detection was independently associated with age (*p* = 0.013) (Table 3). However, this age-related association was only significant during the first month following VGC cessation (*p* < 0.05) and was not maintained throughout the follow-up period (Table 4). Other factors associated with CMV-DNA detection were underlying disease (*p* = 0.003), donor–recipient serological status (*p* < 0.001), and completion of VGC prophylaxis (*p* = 0.002). No significant differences in CMV-DNA detection were observed based on gender (*p* = 0.308) or type of transplant (*p* = 0.807) (Table 3).

Lymphocyte counts were significantly lower in patients with CMV-DNA detection at days 60 and 90 after the discontinuation of VGC (*p* = 0.05 and *p* = 0.01, respectively). Tacrolimus levels tended to be higher in the group of patients with CMV-DNA detection but did not reach statistical significance. No significant differences in mycophenolate levels were observed between patients with and without CMV-DNA detection (Table 4).

The risk of CMV infection was significantly higher in the D+R− patients compared to in the other risk groups (*p* < 0.001) (SHR D+R+: 1.90, CI 95%: 1.31–2.77, *p* = 0.001; SHR D+R−: 5.19, CI 95%: 2.78–9.67, *p* = 0.000). The multivariate analysis showed that the only risk factor associated with CMV-DNA detection was belonging to the high-risk group (odds ratio D+R−: 5.78, *p* = 0.01, 95% CI, 1.32–22.67).

The recipients of organs from seropositive (D+) donors had significantly higher viral loads compared to those receiving organs from seronegative (D−) donors (D+R− hazard ratio (HR): 4.36, 95% CI 2.43–7.84, *p* = 0.000; D+R+ HR: 1.80, 95% CI 1.23–2.65, *p* = 0.003).

**Figure 2:**
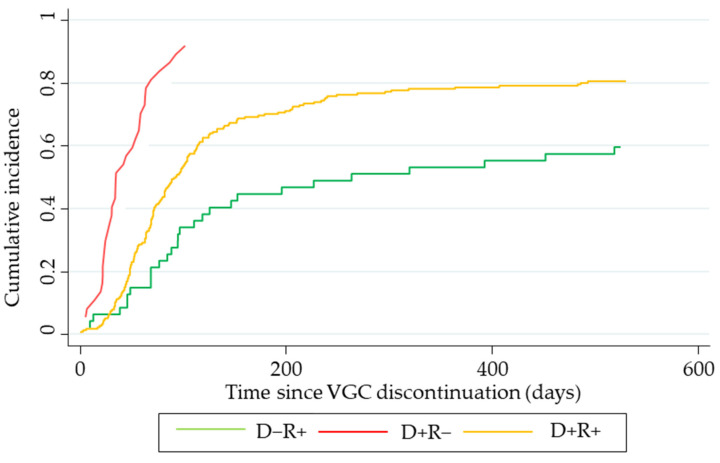
Cumulative incidence curve of CMV-DNA detection.

Figure 2 shows the cumulative incidence (CIF) of CMV-DNA detection after VGC discontinuation for the different risk groups during the 18-month follow-up period. The CIF of CMV-DNA detection for D−R+, D+R+, and D+R− was, respectively, 13%, 28%, and 70% at 60 days and 26%, 49%, and 86% at 90 days since VGC cessation.

The median time from the discontinuation of prophylaxis to the first instance of CMV-DNA detection was 71 days. The D+R− recipients had a significantly shorter time to CMV-DNA detection compared to the R+ ones (D+R−: 35 days, D+R+: 73 days, and D−R+: 96 days; *p* < 0.005).

The patients who did not complete the recommended duration of prophylaxis had significantly earlier episodes of CMV-DNA detection compared to those who completed it (57 days vs. 83 days, *p* = 0.0025).

**Figure 3:**
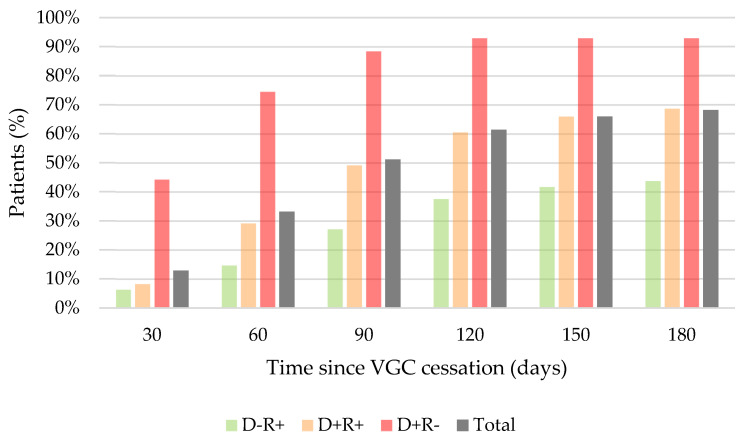
Time from VGC cessation to development of CMV-DNA detection.

Donor CMV-positive (D+) serostatus was significantly associated with the timing of CMV-DNA detection across the different time periods analyzed (*p* < 0.001) (Table 5, Figure 3). At 30 days after VGC discontinuation, 44% (n = 19/43) of the D+R− recipients had had at least one episode of CMV-DNA detection.

## 4. Discussion

Extended antiviral prophylaxis protocols with VGC have reduced CMV-associated morbidity and mortality in lung transplant recipients [9,20]. However, the prolonged administration of VGC recommended by international guidelines may be associated with a higher likelihood of adverse reactions. In this study, we have observed that the discontinuation of VGC due to adverse effects was very frequent in LTx. Our results also show that the discontinuation of VGC was associated with a high frequency of CMV-DNA detection, especially in high-risk patients.

The CMV prophylaxis protocol applied in our Lung Transplant Unit is consistent with the guideline-recommended duration of 6–12 months [9,21,22,23]. However, prophylaxis was prematurely discontinued by almost half of our patients due to the development of side effects. The discontinuation rate observed in our study is similar to the 50% reported by Wiita et al. in a cohort of 123 lung recipients [24], but higher than the 23% reported by Khurana et al. in their sample of 106 patients [17]. These differences are partly due to the designs of VGC prophylaxis regimens, the duration of which was limited to 90 days in Khurana’s study [17], ranged from 6 to 12 months in our protocol, and was indefinite in Wiita et al.’s study [24].

The main causes of discontinuation in our patients were neutropenia, deterioration of renal function, and digestive intolerance. Similar results have been described by other authors [14,24,25,26]. LTx are particularly susceptible to the development of neutropenia as a result of the co-administration of immunosuppressants, such as azathioprine or mycophenolate, and antimicrobials, such as VGC and trimethoprim–sulfamethoxazole. The 43% incidence of neutropenia observed in our patients is similar to the 42% reported by Tague et al. in a cohort of 228 lung transplant recipients from 2008 to 2013 [27].

There are no established protocols for the management of neutropenia in SOT recipients. The discontinuation of mycophenolate and the use of G-CSF have been associated with a higher risk of acute and chronic rejection [27,28]. Our management protocol includes mycophenolate dose reduction or suspension and VGC discontinuation according to the patient’s characteristics and the physician’s criteria. G-CSF is prescribed only in the case of severe neutropenia.

To reduce the incidence of adverse effects of VGC and improve compliance with the drug, some authors have proposed reducing the usual recommended dose of 900 mg/24 h to 450 mg/24 h [21]. Several studies have been performed to describe the benefits of ‘mini-dose’ strategies in kidney transplant recipients [29,30,31,32]. In their sample of high-risk renal recipients, Gabardy et al. reported a higher prevalence of CMVD in the 900 mg dose group vs. the 450 mg one [30]. The authors attributed this finding to the theory proposed by Singh et al. [33] in relation to the low immune response of patients without contact with low viral loads as a result of high doses of VGC.

Kalil et al. conducted a meta-analysis of 12 trials with doses of 900 mg/day versus 8 trials with doses of 450 mg/day performed on SOT recipients. The analysis revealed a significant reduction in the risk of leukopenia and acute rejection in patients receiving a ‘mini dose’ of VGC with equivalent reductions in the frequency of invasive disease [34].

In a retrospective study of LTx, Hunt et al. described lower rates of CMV infection with the standard dose and similar levels of leukopenia in both groups (450 mg vs. 900 mg) [35]. The main limitation for the application of a ‘mini dose’ in lung transplant recipients is the lack of conclusive evidence and the risk of developing resistance to CMV. It is, therefore, not an appropriate recommendation unless blood levels can be monitored [36,37].

Other alternatives for reducing the toxicity associated with prolonged CMV administration include pre-emptive therapy, immune response monitoring, and the use of new drugs such as letermovir. Pre-emptive therapy has the potential advantages of reducing adverse effects, reducing overall costs, and allowing for the development of an immune response to CMV in the absence of initial prophylactic therapy [26,33]. The frequency of CMVD described by Piloni et al. in 129 lung transplant recipients managed with the CMV pre-emptive therapy approach was 5% and was lower than that reported in other studies [38,39,40]. Letermovir is an oral alternative with comparable efficacy and lower toxicity than VGC. In high-risk kidney transplant patients with neutropenia or difficulties managing VGC, letermovir can be used as an alternative [10,41]. Several studies have demonstrated its safety and tolerability [41,42], but the absence of prospective trials in LTx prevents its general adoption as a first-line agent for CMV prophylaxis in these patients.

VGC prophylaxis does not prevent late replication after the discontinuation of prophylaxis. In our study, 79% of the patients who completed the follow-up had at least one episode of CMV-DNA detection after the cessation of VGC. Our detection rate was higher than that reported by other authors [43,44]. Revuelta et al., in a cohort of 239 patients with demographic characteristics similar to ours, described 88 events (37%) related to CMV: 65 (73.9%) infection events, 11 (12.5%) syndrome events, and 12 (13.6%) disease events [44]. Despite the high frequency of CMV-DNA detection in our study, it is important to highlight that over 70% of the patients with detected CMV-DNA had low viral loads. These results are similar to those of Chang et al., who observed that most cases of infection had low viral load detection (<10^3^ copies/mL) [43]. The higher frequency observed in our study may be partly due to more thorough monitoring, the use of more sensitive tests, and the inclusion of all positive results, including those of ≤35 IU/mL. This viral load level was chosen because it is the one used in our medical practice as a warning for closer follow-up.

The clinical significance of low CMV-DNA levels is difficult to interpret. Understanding viral kinetics is essential to identify patients at risk of developing CMVD [45]. Low levels of CMV-DNA may indicate ongoing active infection and a risk of progression to high viral loads and disease, especially in high-risk recipients [46]. This is because the rate of CMV replication is significantly faster in naive recipients than in R+ ones [47]. Some patients have transient positive PCR results after a previous negative detection. These positive results, referred to as “blips” by some authors, are relatively common and may indicate low levels of viral replication or reflect variability in the technique [48]. Lodding et al. observed that the likelihood that PCR detection represents a true ‘blip’ decreases in the presence of high viral loads and increases in low- and intermediate-risk recipients. The same authors had previously reported that the viral load doubling time can be as short as 4 days, regardless of the type of transplant and the serostatus between the donor and recipient [49]. Therefore, a second test within 4 days may be useful to confirm and/or exclude if the positive test is indeed a ‘blip’. Negative or low viral loads should be interpreted with caution in patients with symptoms, as they do not rule out viral replication at the pulmonary or gastrointestinal level. In this regard, it is important to note that the detection of CMV-DNA does not always translate into clinical disease but could indicate a potential risk that requires short-term follow-up.

The median time from the discontinuation of VGC to the first instance of detection of CMV-DNA was 71 days for all patients who completed the follow-up. However, in high-risk recipients, we observed a significantly lower median time of 35 days. It is difficult to compare our results with those previously described by other authors because most publications describe the time to the first instance of detection of CMV-DNA in relation to the date of transplantation and not from the date of VGC suppression. In addition, the duration of prophylaxis can be variable and has not been well defined in some reviews. Chang et al. described a median time from transplantation to viremia of 665 days, with times of more than 12 months in D+R− recipients, 203 days in the D−R+ recipients, and 202 days in the D+R+ ones [43]. However, in some patients, the authors could not accurately determine the duration of prophylaxis.

The development of viral replication after suppression of VGC may be influenced by the duration of prophylaxis. Similar to other reports, we observed that patients who did not complete prophylaxis presented more frequently viral detection (87% vs. 73%; *p* = 0.002). In a retrospective review, Palmer et al. found that the incidence of CMV infection decreased significantly after increasing the duration of prophylaxis from three to twelve months (64% vs. 10%; *p* = 0.001) [14]. The importance of prophylaxis duration was also highlighted by Zamora et al. in a prospective study of recipients at risk (D+R− and R+) in which they observed that patients who received 180, 270 or 365 days of prophylaxis with VGC had significantly lower rates of CMV infection and disease than patients who received short-course prophylaxis [50].

Pre-transplant serostatus, as a risk factor for CMV infection, has already been reported [6,51]. Organ recipients with no immunity (R−) in whom primary infection is triggered by receiving the organ from a seropositive donor (D+) are at the highest risk. R+ patients who receive an organ from a seropositive donor of a different strain (D+R+) are also at high risk. These patients have almost twice the risk of infection as positive recipients receiving a negative organ (D−R+). Consistent with previous observations, in our study, D+R− and D+R+ patients were more likely than D−R+ patients to have CMV-DNA detected after VGC suppression (D+R−: 93%; D+R+: 69%; D−R+: 44%).

Regardless of the type and duration of prophylaxis, some authors have reported that CMV transmission from a seropositive donor to a seronegative recipient is higher in lung transplant recipients than in liver, heart, kidney, or pancreas recipients [6,9,13]. In our study, we observed that receiving a graft from a positive donor was associated with an increased risk of viral detection, regardless of the recipient’s serostatus. In this regard, macrophages transferred with the seropositive graft may act as a long-term reservoir of potential reactivation, and the duration of their survival may be longer than the duration of most current antiviral prophylaxis regimens [52]. Therefore, it is recommended that the designs of future studies take into account CMV transmission criteria, especially in high-risk organ recipients (D+R−).

Cell-mediated immune response is the primary defense mechanism against CMV. Lymphocytes are critical in controlling viral replication and preventing infection. We have observed that peripheral blood lymphocyte counts on days 60 and 90 after VGC suppression were significantly associated with CMV-DNA detection. These results are consistent with the increased likelihood of invasive disease or relapse described by other authors [53,54].

The significant age differences observed among the three pre-transplant serostatus groups could be attributed to the increase in CMV seroprevalence associated with age, as described in the general population [55]. The older age of patients with post-transplant CMV-DNA detection observed in our study may be attributed to both age-related immunosenescence and the increased likelihood of older donor lungs harboring latent CMV [51,56,57].

### Limitations and Suggestions

The major limitation of this study is its retrospective, single-center nature. Additionally, the 18-month follow-up period may not fully capture long-term outcomes. However, we consider this period to have the highest risk of CMV detection. Extending the follow-up beyond 18 months after prophylaxis discontinuation could be of interest and a matter of consideration for future studies.

## 5. Conclusions

In conclusion, the discontinuation of VGC due to adverse events is very frequent in lung recipients. Patients who fail to complete the recommended duration of prophylaxis experience earlier and more frequent viral replication. CMV-DNA detection is very common after the discontinuation of VGC and is related to CMV donor and recipient serostatus. Physicians should be aware of the risk of infection following VGC cessation and ensure close post-prophylaxis monitoring of CMV-DNA, tailored to the patient’s characteristics and viral dynamics.

## Figures and Tables

**Table 1:** Demographic characteristics of the cohort.

	D+R+	D−R+	D+R−	Total	*p* Value
**Total (n, %)**	221 (71%)	48 (15%)	43 (14%)	312 (100%)	
**Age (mean ± SD)**	54.65 ± 11.51	56.29 ± 9.58	44.47 ± 14.80	53.50 ± 12.27	0.000 ^1^
**Gender (n, %)**					0.247
Male	137 (62%)	36 (75%)	28 (65%)	201 (64%)	
Female	84 (38%)	12 (25%)	15 (35%)	111 (36%)	
**Type of transplant (n, %)**					0.283
Bilateral lung	177 (80%)	35 (73%)	37 (86%)	249 (80%)	
Unilateral lung	44 (20%)	13 (27%)	6 (14%)	63 (20%)	
**Underlying disease (n, %)**					0.067
COPD/emphysema	101 (46%)	25 (52%)	15 (35%)	141 (45%)	
ILD	84 (38%)	18 (38%)	13 (30%)	115 (37%)	
Cystic fibrosis	25 (11%)	4 (8%)	12 (28%)	41 (13%)	
Others	11 (5%)	1 (2%)	3 (7%)	15 (5%)	

^1^ Significant differences were observed between the age groups (*p* < 0.001), particularly between D+R− and D−R+ (*p* < 0.001) and between D+R− and D+R+ (*p* < 0.001). However, no significant differences were found between D+R+ and D−R+ (*p* = 1.000).

**Table 2:** Demographic characteristics in terms of VGC discontinuation.

	VGC Completed	VGC Not Completed	Total	*p* Value
**Total (n, %)** **^1^**	179 (57%)	133 (43%)	312 (100%)	
**Age (mean ± SD)**	52.5 ± 12.40	54.87 ± 12		*p* = 0.088
**Gender (n, %) ^1^**				*p* = 0.379
Male	119 (59%)	82 (41%)	201 (100%)	
Female	60 (54%)	51 (46%)	111 (100%)	
**Underlying disease (n, %) ^1^**				*p* = 0.054
COPD/emphysema	72 (51%)	69 (49%)	141 (100%)	
ILD	74 (64%)	41 (36%)	115 (100%)	
Cystic fibrosis	27 (66%)	14 (34%)	41 (100%)	
Others	6 (40%)	9 (60%)	3 (100%)	
**Type of transplant (n, %) ^1^**				*p* = 0.807
Bilateral lung	37 (59%)	26 (41%)	63 (100%)	
Unilateral lung	142 (57%)	107 (43%)	249 (100%)	
**Donor (D)/Recipient (R) serostatus (n, %) ^1^**				*p* = 0.003
D+R−	13 (30%)	30 (70%)	43 (100%)	
D+R+	137 (62%)	84 (38%)	221 (100%)	
D−R+	28 (58%)	20 (42%)	48 (100%)	

^1^ Expressed as the number of patients who completed the recommended duration of VGC prophylaxis vs. those who discontinued it prematurely.

**Table 3:** Demographic characteristics of patients in terms of CMV-DNA detection.

	CMV-DNA Detection	No CMV-DNA Detection	*p* Value
**Total (n, %)**	246 (79%)	66 (21%)	
**Age (mean ± SD)**	54.39 ± 11.60	50.16 ± 14.13	*p* = 0.013
**Gender (n, %)**			*p* = 0.308
Male	162 (81%)	39 (19%)	
Female	84 (76%)	27 (24%)	
**Type of transplant (n, %)**			*p* = 0.647
Bilateral lung	195 (79%)	54 (82%)	
Single lung	51 (81%)	12 (19%)	
**Underlying disease (n, %)**			*p* = 0.003
COPD/emphysema	124 (88%)	17 (12%)	
ILD	83 (72%)	32 (28%)	
Cystic fibrosis	27 (66%)	14 (34%)	
Others	12 (80%)	3 (20%)	
**Donor (D)/Recipient (R) serostatus (n, %)**			*p* < 0.001
D−R+	29 (60%)	19 (40%)	
D+R+	177 (80%)	44 (20%)	
D+R−	40 (93%)	3 (7%)	
**Complete prophylaxis with VGC** **(n, %)**			*p* = 0.002
Not completed	116 (87%)	17 (13%)	
Completed	130 (73%)	49 (27%)	

**Table 4:** Age, lymphocytes, tacrolimus, and mycophenolate levels after VGC discontinuation.

		Time to CMV-DNA Detection (Days)					
	CMV-DNA Detection	30	60	90	120	150	180
**Age (years)** **(mean ± SD)**	**No**	54.1 ± 11.8 *	53.5 ± 12	53.1 ± 12.50	52.9 ± 12.9	52.89 ± 12.8	52.65 ± 12.8
	**Yes**	49.7 ± 14.9 *	53.4 ± 12.9	53.8 ± 12.10	53.9 ± 11.9	53.8 ± 12	53.90 ± 12
**Lymphocytes** **^ƚ^** **(^10^3^/µL)**	**No**	1.6 (1.1–2.1)	1.66 * (1.1–2.2)	1.73 * (1.2–2.2)	1.69 (1.2–2.2)	1.59 (1.1–2.2)	1.7 (1.2–2.3)
	**Yes**	1.49 (1–2)	1.43 * (1–1.9)	1.46 * (1–1.9)	1.51 (1.1–2)	1.57 (1.1–2)	1.55 (1.1–2)
**Tacrolimus** **^ƚ^** **(ng/mL)**	**No**	10.6 (8.1–13.6)	10.3 (8–13.8)	9.75 (8–13.6)	9.4 (8–12.8)	9.8 (8.2–13.3)	9.7 (8.1–13.4)
	**Yes**	11.8 (9.7–15.1)	11.4 (9–13.7)	11.5 (8.9–14)	11.5 (8.7–14.1)	11.1 (8.5–14.1)	10.95 (8.6–14)
**Mycophenolate** **^ƚ^ (µg/mL)**	**No**	2.8 (2–3.7)	2.8 (2–3.7)	2.9 (2.1–3.7)	2.9 (2.2–3.7)	2.95 (2.4–3.7)	3 (2.3–4.3)
	**Yes**	2.5 (1.2–3.3)	2.7 (1.5–3.8)	2.6 (1.5–3.7)	2.7 (1.6–3.7)	2.7 (1.6–3.7)	2.7 (1.6–3.7)

* Age shows significant differences at day 30 (*p* = 0.04). Lymphocyte levels show significant differences at days 60 (*p* = 0.05) and 90 (*p* = 0.01), while no significant differences are observed for tacrolimus and mycophenolate at any time point. **^ƚ^** p50 (p25–p75).

**Table 5:** Patients with CMV-DNA detection in terms of time since VGC cessation.

Donor (D)/Recipient (R) Serostatus (n, %) ^1^	Time Since VGC Cessation (Days)					
	30 *	60 *	90 *	120 *	150 *	180 *
D−R+	3 (6%)	7 (15%)	13 (27%)	18 (38%)	20 (42%)	21 (44%)
D+R+	18 (8%)	64 (29%)	108 (49%)	133 (60%)	145 (66%)	151 (69%)
D+R−	19 (44%)	32 (74%)	38 (88%)	40 (93%)	40 (93%)	40 (93%)
Total	40 (13%)	103 (33%)	159 (51%)	191 (61%)	205 (66%)	212 (68%)

^1^ Expressed as the number and proportion of patients in the serological subgroup with CMV-DNA detection. * Differences in CMV-DNA detection between groups and times were statistically significant when compared using Pearson’s chi-square test: *p*-values (between groups for each time point) <0.001; *p*-values (between time points for each group) < 0.001.

## Data Availability

The raw data supporting the conclusions of this article will be made available by the authors on request.
